# Spatiotemporal perturbations in paced finger tapping suggest a common mechanism for the processing of time errors

**DOI:** 10.1038/s41598-019-54133-x

**Published:** 2019-11-28

**Authors:** Sabrina Laura López, Rodrigo Laje

**Affiliations:** 1 Sensorimotor Dynamics Lab, Departamento de Ciencia y Tecnología, Universidad Nacional de Quilmes, Bernal, Argentina; 2 CONICET, Buenos Aires, Argentina

**Keywords:** Cognitive neuroscience, Human behaviour, Nonlinear phenomena

## Abstract

Paced finger tapping is a sensorimotor synchronization task where a subject has to keep pace with a metronome while the time differences (asynchronies) between each stimulus and its response are recorded. A usual way to study the underlying error correction mechanism is to perform unexpected temporal perturbations to the stimuli sequence. An overlooked issue is that at the moment of a temporal perturbation two things change: the stimuli period (a parameter) and the asynchrony (a variable). In terms of experimental manipulation, it would be desirable to have separate, independent control of parameter and variable values. In this work we perform paced finger tapping experiments combining simple temporal perturbations (tempo step change) and spatial perturbations with temporal effect (raised or lowered point of contact). In this way we decouple the parameter-and-variable confounding, performing novel perturbations where either the parameter or the variable changes. Our results show nonlinear features like asymmetry and are compatible with a common error correction mechanism for all types of asynchronies. We suggest taking this confounding into account when analyzing perturbations of any kind in finger tapping tasks but also in other areas of sensorimotor synchronization, like music performance experiments and paced walking in gait coordination studies.

## Introduction

Sensorimotor synchronization (SMS), the mainly specifically human ability to keep synchrony with an external periodic metronome^1–4^, is a spontaneous behavior and, despite its simplicity, the mechanisms underlying it remain mostly unknown. The processing of temporal information is an open area of research in neuroscience and how time is represented and manipulated in the brain is still one of the most elusive concepts, particularly in this timing range called *millisecond timing* (hundreds of milliseconds)^5–9^.

**Figure 1:**
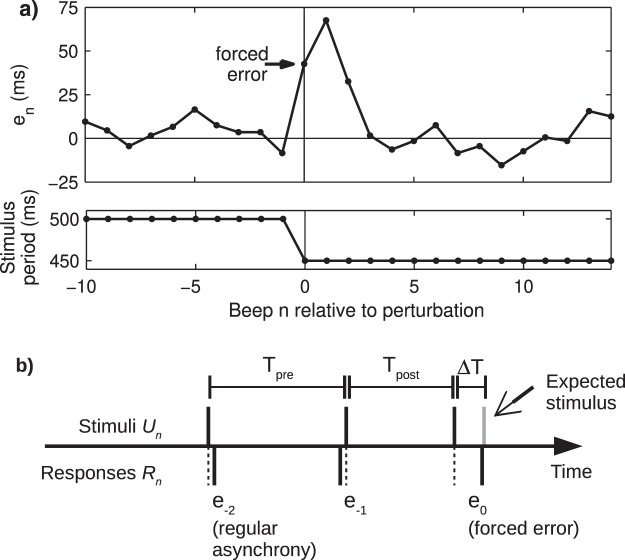
Paced finger tapping task with an unexpected tempo perturbation. (**a**) Asynchrony time series (top) from a single trial from one subject showing the forced error produced by a tempo decrease (bottom) occurring at *n* = 0. The time series was vertically shifted such that its pre-perturbation baseline is zero. (**b**) Schematic and definitions. As the perturbation Δ*T* is unexpected, a change in the variable *e*_0_ occurs in addition to the change in the parameter *T*.

The most used experimental paradigm in SMS is auditorily paced finger tapping, a task where a subject is instructed to move a finger (tap) in synchrony with a periodic stimulus sequence of short tones (beep) as in keeping pace with music (Fig. 1a). The main observable, both for mathematical modeling^1^ and behavior description^10^, is the asynchrony (or synchrony error) *e*_*n*_ = *R*_*n*_ − *U*_*n*_, that is the difference between the occurrence time of each response *R*_*n*_ and the occurrence time of its corresponding stimulus *U*_*n*_. Under isochronous (constant period) conditions *e*_*n*_ has a mean and a standard deviation of a few tens of milliseconds; taps typically precede beeps and thus the average *e*_*n*_ is negative (called negative mean asynchrony, NMA^11^). Subjects are normally able to keep average synchrony, meaning that although *e*_*n*_ is almost never zero, the asynchrony time series remains bounded. Asynchrony data from isochronous conditions are usually analyzed by their autocorrelation/autocovariance function at lag *k*^12,13^ commonly assuming stationarity. We are interested, on the contrary, in the non-stationary data right after a temporal perturbation to the stimuli sequence. Such perturbations trigger a resynchronization behavior allowing the study of the underlying error correction mechanism^11,14^. One of the traditional temporal perturbations is known as a “tempo step change” where the period of the sequence changes abruptly at the perturbation beep (Fig. 1b).

There is, however, an overlooked issue with traditional perturbations. Let’s consider a simple perturbation like the mentioned tempo step change. As the perturbation is unexpected, a forced error occurs (Fig. 1a,b). Specifically, at step *n* when the perturbation occurs both the parameter value (stimuli period *T*) and the variable value (asynchrony *e*_*n*_) change, because the stimulus occurrence time *U*_*n*_ changes and thus *e*_*n*_ changes instantaneously and arbitrarily—importantly, without involving any dynamics of the underlying system. The critical difference between period and asynchrony is that the former has no dynamics by itself (parameter) and its value is up to the experimenter, while the latter is subject to the dynamics of the system under study (variable); they might have, for instance, different neural correlates. Note that this issue appears in any traditional perturbation that modifies any stimulus occurrence in the sequence, whether it be modifying all intervals from the perturbation on (like for instance the above-named tempo step changes^15,16^, but also sigmoidal^17–19^, linear^20^, quadratic^21^, sinusoidal^22,23^, random^22,24–26^, and adaptively timed sequences^27–29^) or a single interval (known as phase shifts^30^), or a single stimulus (known as event onset shifts^31–33^). Up to our knowledge, this is the first work to note this issue.

Beyond the finger tapping literature, research in other areas within SMS are quite likely impacted by this parameter-variable confounding. Two areas are easily spotted. First, research on music performance and perception also relies on perturbation experiments and ecological experimental conditions with natural variability in the stimuli, like accelerando, ritardando, and natural expression^34^, groove and microtiming^35^, musicians following a conductor^36^, phase shift and tempo perturbations^37–39^, and auditory delayed feedback^40^; theoretical works about oscillator models of synchronization to a musical beat^41^ also consider perturbations. Second, research on gait coordination also makes use of experimental designs that might be confound parameter and variable perturbations, like phase shift perturbations^42,43^, interpersonal coordination during side-by-side walking^44^, and varying belt speeds and directions in treadmill walking^45^.

An analogy may help convey our point regarding the difference between parameters and variables. Let’s consider the circadian clock, a well-characterized genetic oscillator that in mammals is located in the suprachiasmatic nucleus of the hypothalamus. The circadian clock displays an autonomous oscillatory activity with a period of around 24 hs capable of synchronizing to the daily cycle mainly through the light/dark stimulus^46,47^. In a minimalistic description, the system works as follows: a gene produces a protein (BMAL/CLOCK) that activates a second gene, which in turn produces a second protein (PER/CRY) that inactivates the first gene. In this system the period of the external light/dark stimulus is a parameter, while the concentrations of CLOCK/BMAL and PER/CRY proteins are the variables whose time evolution is set by the dynamics of the system. Both the parameter and the variables may be experimentally manipulated independently: the parameter value can be modified by changing the period of the external light/dark stimulus (called T-cycle^48,49^), and the value of the variables can be modified for instance by applying an acute dose of inhibitor PF-670462 that modifies the concentration of PER^50^. We do not attempt to make any analogy between physiological and psychological phenomena; we only consider the circadian clock to illustrate that parameters do not have their own dynamics (period of the light/dark cycle in circadian clock – stimulus period in SMS), whereas variables evolve under the system’s dynamics (PER concentration in Circadian Clock – asynchrony in SMS).

It would be desirable in paced finger tapping to manipulate parameter and variable values independently. In this work we perform paced finger tapping experiments with simple temporal perturbations (traditional tempo step changes), and also novel spatial perturbations with temporal effect (raised or lowered point of contact) and combined perturbations. This allows us to decouple the effect of traditional temporal perturbations and perform novel manipulations where the parameter only changes (a change in stimuli period without a change in asynchrony) or where the variable only changes (a change in asynchrony without a change in period). We pursue two questions: whether the underlying error correction mechanism is linear or nonlinear, and whether the mechanism is the same for asynchronies of different origins or not. Our results show nonlinear effects even when the perturbations are 10% of the stimulus period and are compatible with the idea that the origin of the asynchrony does not influence the subsequent resynchronization. The issue of whether motor timing and sensory timing depend on the same neural circuits is still open^7^, but our results suggest that asynchronies produced by purely temporal perturbations and those produced by spatiotemporal perturbations are processed by a common error correction mechanism.

## Results

We performed an auditorily paced finger tapping experiment with perturbations, where the subject is instructed to keep in synchrony at his/her best and keep tapping to resynchronize in case a perturbation appears at a random beep. Perturbations can be any of the following: (a) simple temporal perturbations “T” (traditional tempo step change perturbations by an amount ±Δ*T*); (b) simple spatial perturbations “S” that have temporal effect, achieved by raising (+) or lowering (−) the point of contact where the subject has to tap and thus advancing or delaying the time of contact; c) combined simultaneous perturbations “ST”. Perturbations were classified according to two criteria: by size (small and large perturbations), and by type (simple temporal +/−T; simple spatial + /−S; combined analogous +S+T and −S−T; and combined opposite +S−T and −S+T). Note that in the simple spatial +/−S perturbations the stimuli period doesn’t change and thus they are variable-only perturbations; in the combined opposite +S−T and −S+T the stimuli period changes but the asynchrony doesn’t because both components compensate each other on average at the perturbation beep, and thus they are parameter-only perturbations. See Methods for a detailed description and rationale.

**Figure 2:**
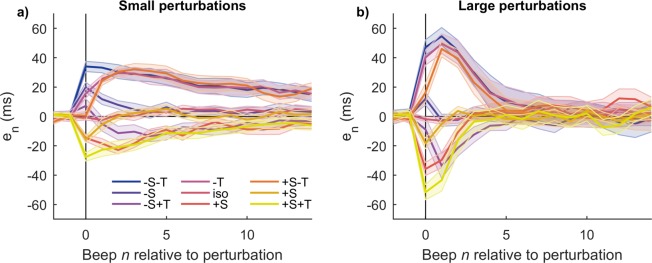
Resynchronization time series for every condition averaged across subjects. Perturbation occurs at *n* = 0. Single trials were vertically shifted before averaging such that its pre-perturbation baseline is zero. (**a**) Small perturbations. (**b**) Large perturbations. All traces are mean +/− standard error across subjects.

Figure 2 shows the average time series for every condition. As the perturbations are unexpected, at the perturbation beep *n* = 0 a forced error occurs for all conditions but +S−T, −S+T, and isochronous. Small perturbations take more steps to recover than large perturbations.

One of the effects of a tempo step change perturbation in paced finger tapping is a shift in baseline relative to its pre-perturbation value^14^. Such baseline is the extensively studied “negative mean asynchrony” or NMA (as defined in the Introduction), the approximately constant average asynchrony value thought to represent the point of subjective synchrony^11^. In this work, on the contrary, we are interested in analyzing the system’s dynamics right after being perturbed and how it converges to its post-perturbation value^14^. In order to do that, from here on we choose to plot every time series after subtracting its post-perturbation baseline such that all time series converge to zero after perturbation (see Methods).

We look for nonlinear signatures in the data, specifically asymmetric responses and non-additivity. If the response to symmetric perturbations is not symmetric, or if compounded perturbations lead to non-additive responses, then the underlying mechanism may not be well represented by a linear model (i.e. a model with only linear terms; examples of linear models are discussed for instance in sections 5 and 6 of ref. ^11^). Nonlinearity can also be seen as saturation effects, or even as the need for separate fitting of different perturbations sizes (and thus separate sets of parameter values) when a linear model is used to describe the data. For a more detailed description of nonlinear effects see ref. ^14^.

### The response to simple perturbations is asymmetric

**Figure 3:**
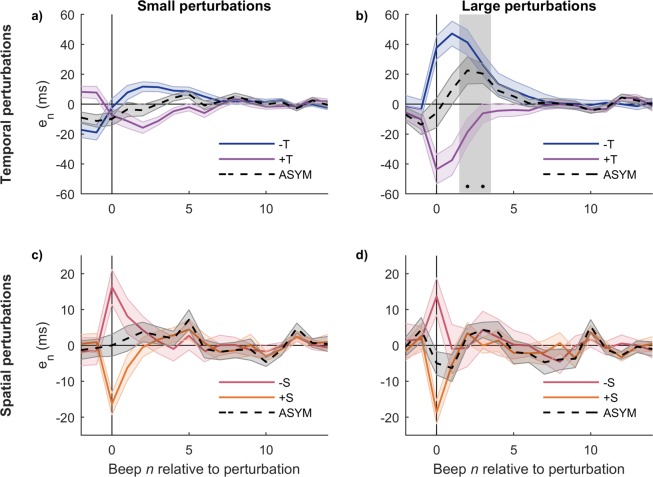
The response to large temporal perturbations is asymmetric. (**a,b**) Response to traditional tempo step change perturbations (+/−T) and degree of asymmetry between them (ASYM). (**c,d**) Response to novel spatial perturbations (+/−S) and degree of asymmetry between them (ASYM). Left: small perturbations; Right: large perturbations. Large temporal perturbations produce asymmetric responses, and the grey vertical rectangle indicates steps where the asymmetry is significative (*p* = 0.0025 each). All traces are mean +/− standard error across subjects.

In Fig. 3a,b we show the averaged time series for perturbations +T and −T. Large perturbations produce asymmetric responses—the response to the positive perturbation converges to zero more rapidly than the negative one, in accordance with previous reports for this type of perturbation^14,16^. Small perturbations, on the other hand, do not show asymmetry^14^. Both responses are consistent with a nonlinear underlying system, where nonlinear behavior like asymmetry is evident only when the perturbation size is large enough^20^.

Spatial perturbations +S and −S, on the contrary, produce responses that are mostly symmetric (Fig. 3c,d). It is worth noting that the asynchrony values produced by +/−S perturbations (both small and large) at the perturbation beep are relatively small and similar in size to those produced by the small +/−T perturbations, which also have symmetric responses (Fig. 3a). The similarity between the responses to the nominally small and large S perturbations might be a limitation of our current setup, and is addressed in the Discussion. Spatial perturbations +/−S are novel in that they produce a change in the value of the variable (asynchrony *e*_*n*_) without changing the stimulus period.

### The response to combined perturbations is asymmetric

**Figure 4:**
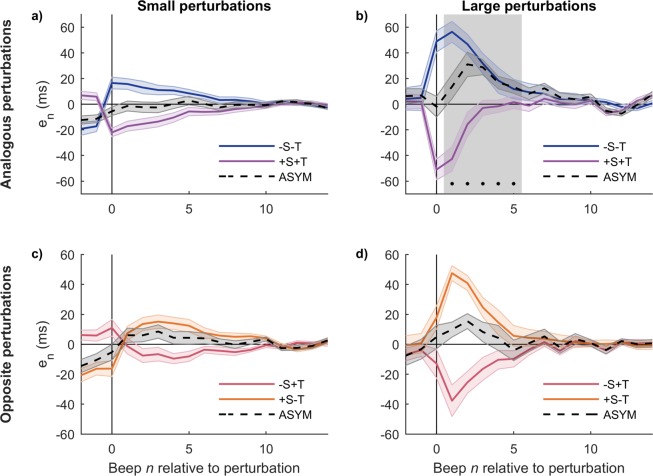
Large combined perturbations produce asymmetric responses. (**a,b**) Response to analogous perturbations −S−T and +S+T (whose components S and T would individually produce asynchronies of the same sign) and degree of asymmetry (ASYM). (**b,c**) Response to opposite −S+T and +S−T (whose components S and T would individually produce asynchronies of opposite signs) and degree of asymmetry (ASYM). Left: small perturbations; Right: large perturbations. When compared to the large temporal perturbations of the previous section, analogous perturbations produce more asymmetric responses while opposite perturbations produce less asymmetric responses. Mean +/− standard error across subjects. The grey vertical rectangle indicates steps when the asymmetry is statistically significant (*p* = 0.022, 0.0025, 0.0025, 0.0067, 0.022, respectively).

In this section we analyze the degree of asymmetry of the combined perturbations, and for that we group them in “analogous” (+S+T and −S−T, because their components S and T individually would produce asynchronies of the same sign) and “opposite” (+S−T and −S+T, whose components would individually produce asynchronies of opposite signs; see Methods). Figure 4 shows that the large analogous perturbations produce asymmetric responses (positive ASYM, statistically significant), while in the large opposite perturbations ASYM is also positive but fails to reach significance. When compared to the large temporal perturbations (+T y −T, Fig. 3) the degree of asymmetry seems greater in the analogous perturbations (−S−T and + S + T) and lesser in the opposite perturbations (−S+T and +S−T).

In order to interpret this result, we note that the analogous perturbations in Fig. 4 produce asynchronies with values at the perturbation beep (*n* = 0) greater than the temporal perturbations, while the opposite perturbations produce values lesser than the temporal perturbations (Wilcoxon sign-rank test, significant after Bonferroni correction; simple temporal vs. analogous *p* = 0.013; simple temporal vs. opposite *p* = 0.00073; analogous vs. opposite *p* = 0.00048). The result in this section may then be understood within the same framework that the result from the previous section: it is the behavior of a nonlinear underlying system whose asymmetric response is only evident when the asynchrony values are large enough, and whose degree of asymmetry is larger for greater asynchronies.

### Simple perturbations are additive

One of the aims for proposing the combined perturbations (+/−)S(+/−)T was to compare every combination to the simple perturbations it consists of. For example, and to show the notation, we would like to compare between the actual combined perturbation +S−T and the sum of the simple perturbations (+S)+(−T).

**Figure 5:**
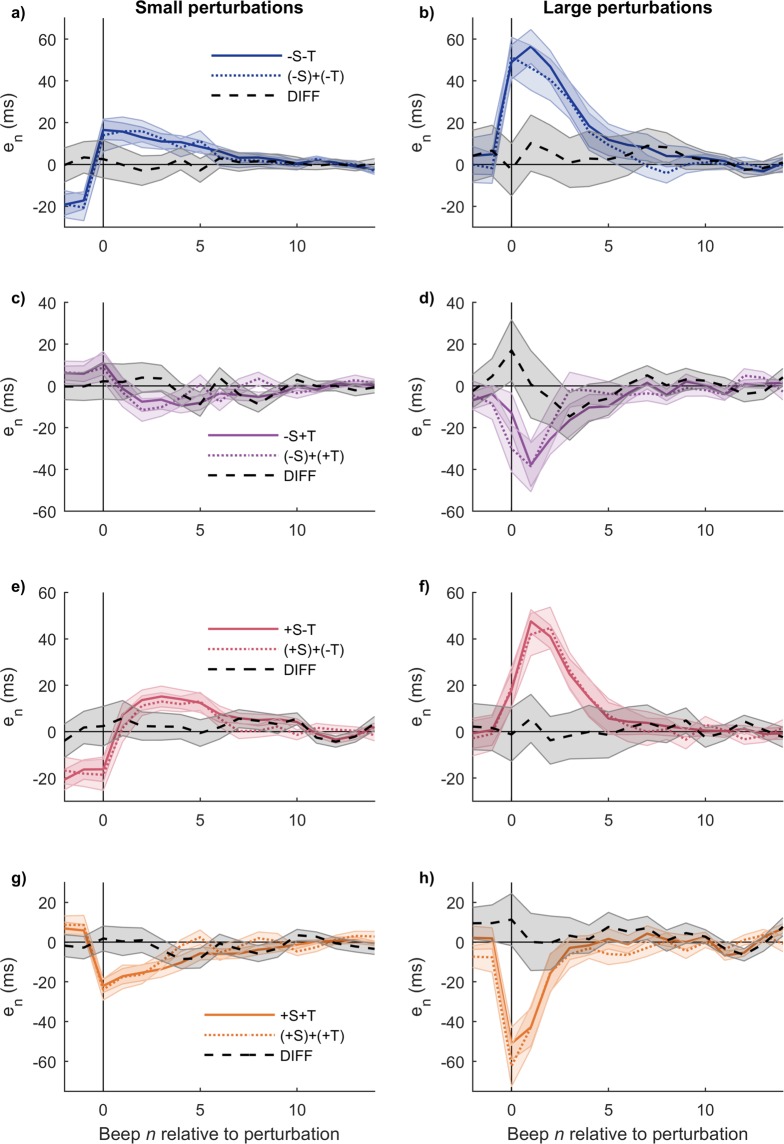
Simple perturbations are additive. Each panel displays the actual combined perturbation, the sum of its individual components, and the difference (DIFF) between the two. (**a,b**) Combined −S−T and sum (−S) +(−T). (**c,d**) Combined −S+T and sum (−S)+(+T). (**e,f**) Combined +S−T and sum (+S)+(−T). (**g,h**) Combined +S+T and sum (+S)+(+T). Left: small perturbations; Right: large perturbations. The DIFF series are not significantly different from zero in any case. Mean +/− standard error across subjects.

Figure 5 shows all comparisons. In each case, the actual combined perturbation elicits responses very similar to the sum of responses of the corresponding simple perturbations, both for small and large perturbations (no significant differences in any case). This result, typically associated with linear systems, is relevant to build a conceptual model of the error correction mechanism in a way consistent with the previous sections.

### Combined perturbations and exploration of new system states

#### Experimental phase space

In a previous work, we showed experimental and theoretical evidence suggesting that the correction mechanism underlying resynchronization after a T perturbation in paced finger tapping is nonlinear and should be described by two variables^14^. If one of the variables of such a system is the observable *e*_*n*_, how could we obtain experimental information about the other variable? The embedding technique^51^ allows us to get information about the geometric organization of the underlying system’s trajectories by analyzing the time series of a single observable, in this case *e*_*n*_, and reconstructing from it an experimental phase space.

Every system has a unique geometrical representation in phase space; that is, a geometrical signature given by the number and type of fixed points, the way the trajectories converge to or diverge from one another, etc^51–53^. Two different systems might have similar signatures—then they are equivalent and can be morphed into one another. Conversely, if two sets of trajectories are not geometrically compatible (e.g. trajectories from different sets cross transversely, or display a different number of fixed points, etc.) then the two sets correspond to different, i.e. non equivalent, systems. For instance, if asynchronies from T and S perturbations were processed by separate mechanisms, we would not expect the trajectories in phase space to be compatible, qualitatively similar or equivalent.

**Figure 6:**
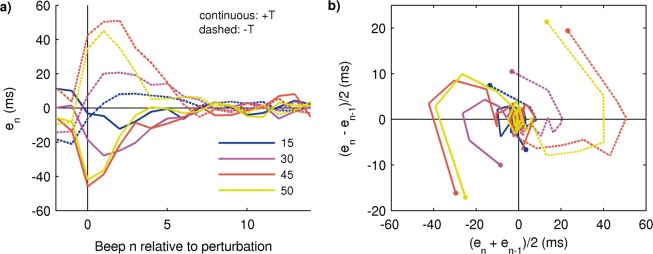
Experimental reconstruction of the phase space by means of an embedding. (**a**) Averaged time series of *e*_*n*_ in response to temporal perturbations T of all sizes and signs. (**b**) Embedding of the time series shown in (**a**). The trajectories are organized in phase space according to the size and sign of perturbation (the smallest perturbations ±15 ms follow the same organization than the larger ones but are visually masked by the post-perturbation variability). The beginning of each trajectory is marked by a dot and corresponds to the perturbation step *n* = 0 in the time series (steps before the perturbation are not shown for visual clarity). All trajectories converge to the origin, representing the post-perturbation baseline. Mean across subjects; the error bands are not shown for visual clarity.

To illustrate the procedure, in Fig. 6 we undo the “small/large” classification of perturbation sizes and show the averaged time series corresponding to the simple temporal perturbations T of all sizes and signs (±15 ms, ±30 ms, ±45 ms, ±50 ms; panel A). In addition, we show in panel B the trajectories that result after reconstruction of the experimental phase space by means of an embedding where the first component is (*e*_*n*_ + *e*_*n*−1_)/2 and the second one is (*e*_*n*_ − *e*_*n*−1_)/2. The perturbation step, *n* = 0 in the time series, corresponds to the initial dot in the trajectory in the embedding, and the origin represents the stationary solution after resynchronization where all asynchronies are zero on average. The trajectories are neatly organized according to size and sign of the perturbation. Responses to larger perturbations begin farther away from the origin; the trajectories for the 45 ms and 50 ms perturbations are very similar as expected by the similitude between the perturbation sizes. The clear geometric organization of the embedded trajectories supports the idea of a common error correction mechanism in charge of resynchronization whose response depends on the size and sign of the perturbation^14^.

#### Trajectories of the combined perturbations

We’ve shown that the simple perturbations S produce a novel effect: a change in asynchrony without a change in the stimulus period. On the other hand, the combined opposite perturbations (+S−T and −S+T) are also novel: they produce a change in the period without changing on average the expected asynchrony value (see Methods and Supplementary Fig. S1). For instance, let’s consider a traditional simple temporal perturbation −T representing a tempo step decrease (a change in stimulus period by an amount −Δ*T*). It normally produces an increase in asynchrony by an amount +Δ*T* at the perturbation step. By combining −T with +S to get a +S−T perturbation, a decrease in stimulus period is produced due to the −T component but with a lesser asynchrony value because of the temporal compensation produced by the +S component. Conversely, the combined analog perturbations (+S+T and −S−T) make the asynchrony value greater (in absolute value) than the one produced by a T perturbation alone.

**Figure 7:**
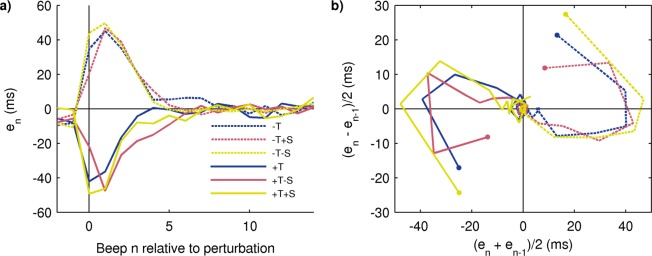
Response to combined perturbations of ±50 ms and experimental reconstruction of phase space. (**a**) Averaged time series of traditional simple temporal perturbations (+T and −T), combined opposite perturbations (−S+T and +S−T), and combined analogous perturbations (+S+T and −S−T). (**b**) Embedding of the time series shown in (**A**); the dots correspond to the perturbation step (*n* = 0; previous steps are not shown for visual clarity). The paths taken by the opposite and analogous perturbations are very similar to the simple temporal perturbations but with different initial points; the initial points are organized according to the expected asynchrony value (from the origin outwards: opposite-simple-analogous). Mean across subjects; the error bands are not shown for visual clarity.

In Fig. 7a we compare the time series of the traditional perturbations −T and +T (size 50 ms only) and the corresponding combined opposite and analogous perturbations. All three types of perturbations (traditional, opposite, analogous) produce similar time evolutions except for their initial asynchrony value (*n* = 0). In the phase space, this can be seen as the three trajectories on the same side making similar paths after a quick convergence from differing initial points. That is, the responses to perturbations that have a change in period are similar despite different initial asynchrony values.

### All perturbations: new system states

In this section we continue analyzing the experimental phase space shown above by including the information from the novel perturbations presented in this work. The combined perturbations, as well as the already discussed simple spatial perturbations S, allow us to decouple the effect of traditional temporal perturbations and access as yet unexplored system states. We will normalize the trajectories from all perturbations so we can make evident their positions in phase space relative to one another.

**Figure 8:**
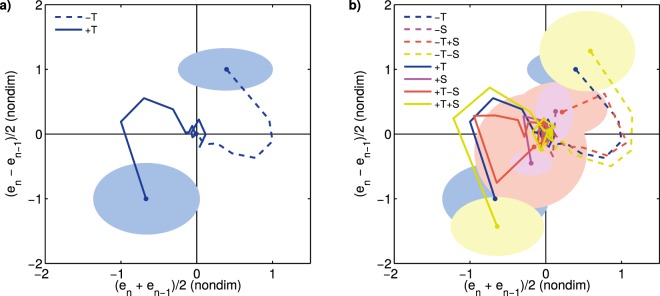
Access to previously unexplored system states. (**a**) Embedding of the responses to traditional perturbations +/− T in normalized, non-dimensionalized, averaged coordinates (as before, the displayed trajectories start at the perturbation step marked by a dot, corresponding to *n* = 0 in the time series). The shaded areas correspond to the region in phase space where the system is left at the perturbation step (center: mean across subjects; radii: standard deviation across subjects). (**b**) Embedding of all perturbations. The novel perturbations probe the system in new ways by setting initial conditions (i.e. at the time of perturbation) never tested before; this is evidenced by the areas corresponding to novel perturbations only partially overlapping with the traditional perturbation.

Figure 8a shows the average of simple temporal perturbations +/−T (averaging across 45 and 50 ms sizes only for clarity, and after normalization and non-dimensionalization; see Methods). Shaded areas represent the regions in phase space (i.e. system states) explored by the traditional tempo step change perturbations. In Fig. 8b we include the rest of the perturbations (simple spatial +/−S, combined opposite +/−S−/+T, and combined analogous +/−S+/−T, after normalization and non-dimensionalization). It is evident that the regions in phase space explored by the novel perturbations overlap with the traditional +/−T but only partially, and thus they allow us to probe the system in novel ways and access system states as yet unexplored as a result of decoupling the experimental manipulation of period and asynchrony.

### A coarse-grained conceptual model for the error correction mechanism

The results shown in the previous sections allow us to distinguish between various ways of how temporal information is processed in this task. We will consider a very simple, coarse-grained, conceptual model for the error correction mechanism. The input is the temporal information coming from performance monitoring (time perception) and that could enter the system through different paths depending on the condition—for instance, temporal information from a simple temporal perturbation T could travel a different path than the temporal information from a simple spatial perturbation S. On the other end, the output is the result of processing such temporal information and drives the motor output. The central part is the time processing itself where hypothetical processes like time comparison, decision making and time prediction and production take place^5,6,8,9,54–56^, and where the estimation of the appropriate asynchrony correction is made. It should be noted that the parts of this model are not necessarily associated with individual brain regions. The central part of time processing might include sensory components (at its beginning) and motor components (at its end), besides the hypothesized processes for time comparison, decision making, etc. That is, part of the processing itself might take place in sensory and/or motor cortices. We propose the following hypotheses for the error correction mechanism:

1. The processing of temporal information is nonlinear (in particular: the response to symmetric perturbations is not symmetric, and the response to a combined perturbation is not equal to the sum of responses of the individual perturbations).
2. The processing of temporal information coming from temporal perturbations and from spatiotemporal perturbations is performed by a common mechanism.

**Figure 9:**
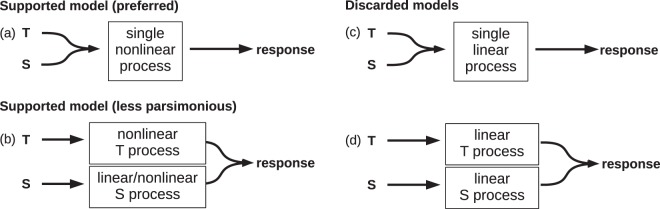
Coarse-grained conceptual models for the processing of temporal information. Models (**a,c**) assume a common mechanism for the processing of temporal information coming from either temporal manipulations T or spatial (spatiotemporal) manipulations S. Models (**b,d**) assume that T and S are processed separately and are added up at the end.

Our hypotheses are represented in four versions of the conceptual model as combinations of 1) linear vs. nonlinear processing, and 2) common vs. separate processing. The four combinations are displayed in Fig. 9. A straightforward result is the observed asymmetric responses, sections “Response to simple perturbations…” and “Response to combined perturbations…”, that validate hypothesis 1 and thus the linear models (c) and (d) must be discarded. This is in agreement with previous reports of nonlinearity, particularly asymmetric behavior (see ref. ^14^. and references therein). It is worth noting that the symmetric responses to the spatial perturbations S (Fig. 3b) don’t necessarily imply that the processing of asynchronies coming from spatial perturbations is linear and consequently discard hypothesis 1—as we noted above, spatial perturbations S (both small and large) produced small asynchronies in comparison to the ones produced by the large temporal perturbations T and thus they might be insufficient to elicit typical nonlinear behaviors.

Now consider specifically the non-additivity aspect of hypothesis 1 together with hypothesis 2. An unwarranted conclusion from the additivity results presented in Fig. 5 would be that T perturbations and S perturbations are processed either by a common linear mechanism or by separate nonlinear mechanisms and then added up. However, in front of the discussion of the previous paragraph, if the S perturbations are indeed too small to elicit nonlinear behavior then we would rather expect they show as additive. That is, the most parsimonious conclusion is that we cannot discard a single nonlinear mechanism and thus we cannot decide whether hypothesis 2 is true or false. The two models backed up by the results considered so far are then (a) and (b). To distinguish between them, we would have to perform perturbations where the asynchronies produced by S perturbations were comparable in size to those produced by the large T perturbations, something that is infeasible with our current setup (due to the high average finger speed of the subjects leading to small asynchronies even at the maximum possible spatial displacement of the contact point). We are developing an improvement to this novel experimental manipulation.

Finally, consider the results from the phase space analysis (Figs. 6b and 7b). Though not a demonstration, the geometrical arrangement of the trajectories in the embedded phase space clearly favors one model over the other. The geometrical arrangement of the trajectories reveals a degree of organization that is hard to see by looking at the time series alone (Fig. 2), where they cross in apparently unpredictable ways. The remarkably consistent behavior across perturbation sizes, perturbation signs, and perturbation types is noteworthy, suggesting there might be a single underlying mechanism in charge of correcting asynchronies regardless of their origin. Taking all evidence into account, the preferred model is (a). On the other hand, model (b), though not discarded, is less parsimonious.

## Discussion

Experimental work on sensorimotor synchronization and, in particular, both paced and unpaced finger tapping dates back to the end of the XIX century^57^, while the first mathematical models were published 50 years ago^22^. Much work has been done in this area of research to advance our knowledge about the underlying mechanism responsible for achieving average synchrony^1,11^. In particular, perturbations to the sequence period are a usual way to probe the system’s inner workings. As we described in this work, these perturbations are in fact confounded parameter and variable manipulations and, up to our knowledge, no published work has addressed this issue.

Our observation applies to any perturbation to the sequence period: local changes like step changes, phase shifts, and event onset shifts, and global changes like accelerando or ritardando, etc. This issue might be important even in the case of a perturbation where the stimulus period does not change, like for instance time-delayed or -advanced auditory feedback from the taps^58–61^. This type of perturbation is not perfectly analogous to our S perturbations because it introduces a dissociation between auditory feedback and proprioceptive and tactile feedback. Thus, unlike the S perturbations, its relationship to the (perturbed) asynchrony value remains to be elucidated.

Reports of nonlinear behavior in SMS are increasingly frequent (see for instance ref. ^14^. and references therein). The finding of specific nonlinear behaviors can for instance help prove or discard particular neural processes involved in the behavior and interactions among them^62^. On the other hand, linear interpretation of a nonlinear behavior can lead to erroneous conclusions. As a very simple example, consider for instance the asymmetric response to the +/−T perturbations and assume they are driven indeed by a single nonlinear underlying mechanism. Imagine we propose, however, a linear model that says perturbations are corrected as a proportion of the observed asynchrony^11^. A parameter of this model would be a coefficient representing that proportion. This model will not be able to fit positive and negative perturbations with a unique set of parameter values. Since the response is asymmetric, the linear model would need two different parameter values for fitting positive and negative perturbations (i.e. different proportions for positive and negative asynchronies)—implying either two different correction mechanisms, or a single mechanism whose parameters can change their value plus an additional control mechanism to choose between the two sets of parameter values. The search for neural correlates of the behavior and underlying cerebral processes and regions would be mislead by this wrong kind of evidence.

We showed that an experimental phase space can be reconstructed from the averaged time series via embedding and that all trajectories (corresponding to either simple or combined perturbations of any size and sign) organize in a geometrically remarkable way. This supports the notion that the underlying system in charge of the error correction can be considered as a single mechanism^14^, as opposed to many different mechanisms that are turned on or off depending on the magnitude, sign, and type of perturbation, a usual—and frequently implicit—assumption in SMS research when perturbation data are considered^16^. Note that this doesn’t imply that the details of such a mechanism are simple—in fact, we expect that the hypothetical processes at play like time perception, comparison, and production interact in complex ways, particularly in this task where the processing of stimuli and the production of timed responses is ongoing and the processes related to step *n* are likely overlapped in time with those from the previous step *n* − 1^62^. Yet, according to our results, it appears that the response of the system is consistent regardless of whether the asynchrony has a purely temporal or spatiotemporal origin, and of the size and sign of the perturbation, thus favoring model (a). However, model (b) was not strictly ruled out; running larger S perturbations, something infeasible with our current setup, would help disambiguate the issue. Work is underway to improve the present experimental manipulation.

Research about how temporal information is processed in the brain has grown very rapidly in the last decade, particularly research related to the neural underlying mechanisms and involved brain regions^9^. Many important issues remain open, like whether timing is centralized and dedicated or distributed or intrinsic; whether it reflects properties of individual neurons or is the emergent behavior of neural networks; whether sensory and motor timing rely on the same circuitry or not; and so on. Our results show that traditional perturbations are confounded parameter-variable manipulations, and that a broader set of perturbations (to either the parameter, the variable, or both) leave the system in different points of the phase space. This suggests revisiting the usual assumptions about perturbations and interpreting results with this distinction in mind. In the music domain, for instance, our results open the possibility that different brain regions and processes might be recruited in front of a perturbation to either the asynchrony or the tempo^4^, and that asynchrony and stimulus period might have different neural representations or correlates. Whether the known associations between functions and regions would split after solving the confounding is yet to be elucidated.

## Methods

### Ethical considerations

Our experimental protocols were designed in accordance with national and international guidelines and were approved by the Ethics Committee of the University of Quilmes. All participants signed an informed consent.

### Task and subjects

The task was an auditorily paced finger tapping that may, at some random point in the series, present a perturbation. The subject was instructed to keep in synchrony at his/her best by using his/her index finger (holding the wrist in place during the experiment) and keep tapping to resynchronize in case a perturbation appears. Each trial was either isochronous (constant stimulus period and fixed point of contact) or perturbed. Perturbations were any of the following three types: a) simple temporal perturbations “T” (traditional tempo step change perturbations); b) simple spatial perturbations “S” that had a temporal effect (see below); c) combined simultaneous perturbations “ST”. Subjects were volunteers; one subject was not able to complete the task, and three subjects were excluded according to the criteria below. The final number of subjects was *N* = 30 (ages 19–40, mean 29.1, 13 female, 28 right-handed, all subjects used their dominant hand).

### Perturbations

We performed three types of perturbations:

1. Simple temporal perturbations +/−T. These perturbations were the traditional tempo step changes where the stimuli period *T* changes once by an amount ±Δ*T*.
2. Simple spatial perturbations +/−S. These novel perturbations consisted in raising (+) or lowering (−) the contact point between finger and sensor, which had the temporal effect of advancing or delaying the time of contact (when the sensor was in its higher or lower position, respectively) and thus made the asynchrony at the perturbed step more negative or more positive, respectively (see Supplementary Fig. S2).
3. Combined perturbations ST, where simultaneous (i.e. at the same step *n*) S and T perturbations take place: +S+T, −S+T,+S−T, −S−T.

In any case the perturbation was unexpected (it occurred at a randomly chosen step).

### Setup

The setup consisted of two interconnected microcontrollers (Arduino Mega) communicating via the Wire library. The “master” microcontroller received instructions from the control script (MATLAB, The MathWorks, Inc) and started a trial (it received trial parameters, generated the sequence of auditory stimuli, sent instructions to the slave microcontroller, registered taps, sent trial data back to the control script). The master microcontroller had a custom-designed shield^62^ for interfacing and signal conditioning (sum and amplification of audio signals, virtual ground, voltage divider for the force sensor, etc.). Every time a tap was detected, auditory feedback was sent as a brief tone. Stimuli and feedback tones were 50-ms duration square waves of 440 Hz (A4) and 587 Hz (around D5) respectively. Visual feedback from the subject’s hand was avoided by means of a blocking screen. The “slave” microcontroller was in charge of producing the spatial perturbations S whenever the master sent the instruction. The slave microcontroller drove a small platform by means of a servo motor (Savox SC-1258TG Super Speed Titanium Gear Standard Digital) and a rotating arm, displacing the platform upwards or downwards along a linear ball bearing. The platform had a force-sensitive resistor (FSR 406, Interlink Electronics) attached on top of it to detect the subject’s response. Sound was played diotically through Sennheiser HD419 headphones, and subjects adjusted sound volume to a comfortable level.

### Experimental design

The combination of temporal perturbations T, spatial perturbations S, and perturbation direction (positive or negative) resulted in 9 experimental conditions: isochronous (no perturbation), 4 simple perturbations (temporal +T and −T and spatial +S and −S), and 4 combined perturbations (+S+T, −S+T, +S−T, −S−T). Each subject participated in a single session with three phases: Introduction, Calibration, Test. Each phase ended when all trials were successfully completed. Each trial consisted of a sequence of 30 auditory stimuli with a period (interstimulus interval) of *T* = 500 ms until a perturbation occurred randomly between the 15th and the 20th stimuli. In the temporal perturbations the period of the sequence was either increased (positive, +T) or decreased (negative, −T). In the spatial perturbations the vertical position of the platform was either moved upwards (positive, +S) or downwards (negative, −S) from its resting position by a distance that depended on the subject (see calibration below) and the new position was kept until the end of the trial. In the combined perturbations both T and S manipulations were performed simultaneously. The introduction phase was an exposure to the task and the 9 experimental conditions (one trial per condition, 9 trials total) to allow the participant to familiarize with the experimental task. The calibration phase consisted of 8 trials with simple spatial perturbations +/−S with the most extreme positions of the platform (±1.5 cm, 16 trials total), so we can estimate the longest temporal effect of the spatial perturbations for each subject. The test phase consisted of 8 trials from each experimental condition (72 trials total), in three blocks separated by short breaks to prevent the participant from becoming tired. Trials were presented in random order within each phase.

### Calibration

The temporal effect of raising or lowering the platform is subject-dependent since every subject moves the finger at its own speed and, thus, a given spatial displacement of the platform is translated to different times for the finger to reach the new contact point. In the calibration phase we estimated for each subject the average time error produced by the unexpected movement of the platform from its resting position to its two most extreme positions (highest 1.5 cm, lowest −1.5 cm).

### Experiments

We performed two experiments:

#### Experiment 1

Matched temporal and spatial perturbations (i.e., the temporal effect of the spatial perturbation is equal to the assigned temporal perturbation size). Subjects were assigned to either one of the groups shown in Table 1, depending on the recorded average time error during the calibration phase.

**Table 1:** Classification by size of Experiment 1 perturbations.

Average time error during calibration of spatial perturbations	Assigned temporal perturbation size during test phase	Number of subjects
Less than 30 ms	15 ms	*N*_1_ = 12
Between 30 ms and 45 ms	30 ms	*N*_2_ = 5
More than 45 ms	45 ms	*N*_3_ = 5

#### Experiment 2

Unmatched temporal and spatial perturbations. Subjects with a large temporal effect in spatial perturbations were very infrequent, so in order to test larger temporal perturbation magnitudes, we created a fourth category with ±50-ms temporal perturbations and ±15-ms spatial perturbations. Number of subjects: *N*_4_ = 8.

### Exclusion criteria

A trial was correct if the subject began tapping before the 6th stimulus and if the subject didn’t miss any response from there on. Each incorrect trial was repeated until completed successfully. Asynchrony outliers: after finishing the experiment, any trial with very large asynchronies (greater than 145 ms in absolute value, with respect to the pre-perturbation baseline) was discarded. Standard deviation outliers: the distribution of the standard deviation of pre-perturbation asynchronies for each subject was computed, and any trial with a standard deviation greater than 1.5 times the interquartile range (IQR) was discarded. After discarding asynchrony and deviation outliers, subjects with less than 4 correct trials for each condition were removed from further analysis (3 subjects, 3 different conditions).

### Data pre-processing

In order to average across trials and subjects, we aligned all trials at the perturbation step (renamed as *n* = 0) and defined the analysis range from *n* = −10 through *n* = 14 such that within that range all subjects have all responses. We defined the trial pre-perturbation baseline as the average of all asynchronies between *n* = −7 and *n* = −1, removing adaptation effects at the beginning of the trial. The trial post-perturbation baseline was the average asynchrony between *n* = 9 and *n* = 14.

### Classification of perturbations

When specifically considering perturbations we leave out the isochronous condition, and thus there are 4 perturbation sizes (15, 30, 45, 50 ms) and 8 conditions (+T, −T, +S, −S, +S+T, −S+T, +S−T, +S+T).

#### According to their size

A posteriori we grouped the data in two categories according to the perturbation size: small and large perturbations. Small perturbations included 15-ms and 30-ms perturbation sizes (*N* = *N*_1_ + *N*_2_ = 17 subjects), while large perturbations included 45-ms and 50-ms sizes (*N* = *N*_3_ + *N*_4_ = 13 subjects).

#### According to their type

According to their origin and expected effect, perturbations can be classified as:

Simple. Temporal +/−T and spatial +/−S.

Combined. Simultaneously changing the period and raising/lowering the point of contact. If the temporal effect of both components is in the same direction (i.e., the asynchrony at the perturbation step either increases or decreases) the perturbation is Analogous (−S−T and +S+T); if the effect goes in opposite directions (for instance one component increases the asynchrony while the other decreases it) the perturbation is Opposite (−S+T and +S−T). See Supplementary Fig. S1.

### Averaging

Time series in Figs. 2 through 7 are grand averages across subjects. Each subject’s average is the mean of all his/her included trials from the corresponding condition.

### Hypothesis testing

#### Asymmetry

In order to quantify the degree of asymmetry between responses to symmetric perturbations we defined the measure ASYM as the sum of the two corresponding time series (i.e. the algebraic difference between one and the opposite of the other). In this way we can compare time series that have opposite signs by definition (because they result from perturbations with opposite signs) and, at the same time, any series is allowed to change sign in the middle without affecting the analysis (it would be artificially rectified if we otherwise used its absolute value). ASYM values close to zero indicate that the compared time series are mostly symmetric; large positive or negative values indicate asymmetry.

#### Additivity

In order to quantify the additivity between responses to simple perturbations we defined the measure DIFF as the difference between the time series of the experimental combined perturbation and the time series obtained by summing the two corresponding individual simple perturbations. For instance, DIFF is the difference between the experimental combined perturbation +S+T and the algebraic sum of the experimental simple perturbations +S and +T. DIFF values close to zero indicate that the combined compared time series are similar; large positive or negative values indicate that the time series are different.

#### False Discovery Rate (FDR) correction

In order to test the statistical significance of ASYM and DIFF we computed p-values for each step between *n* = 1 and *n* = 5 (both included) and then pass the five p-values to the FDR algorithm^63^ with alpha= 0.05. In order to compute the p-values we generated null distributions of ASYM time series and DIFF time series. We illustrate the procedure with an example. To generate the null distribution of ASYM for the small +T/−T comparison (Fig. 3a) we first pooled all trials from all subjects from both conditions +T and −T; then randomly pick 8 trials, averaged them and assigned them to the “surrogate +T” time series of surrogate subject 1; similarly with other 8 trials to the “surrogate −T” of same subject; these surrogate +T and surrogate −T average series were summed to get the surrogate ASYM for this surrogate subject; we then repeated the steps to generate 17 and 13 surrogate subjects for small and large perturbations respectively in order to match the actual number of subjects; the average across surrogate subjects makes one surrogate grand average ASYM time series, and finally repeated the whole procedure 1000 times to get the null distribution of ASYM time series, i.e. the null distribution of ASYM for each step *n*. Since we are interested in the transient part of the resynchronization, we restricted the FDR analysis to the range *n* = 1 through *n* = 5 (*n* = 0 is excluded because it is the perturbation step and the asynchrony there is a forced error; steps *n* > 5 are too close to the post-perturbation baseline). We then compared the true ASYM value at each step between *n* = 1 and *n* = 5 to the null distribution of ASYM at the same step and computed a p-value as the proportion of null ASYM values above the true value or below its opposite (two-tailed). We applied the same procedure to all comparisons between times series in this work.

### Phase space reconstruction (embedding)

#### Regular trajectories

Given a univariate time series *e*_*n*_, there are several possible alternatives to implement time series embedding for phase space reconstruction^51^, like the usual time-delay embedding (*e*_*n*_;*e*_*n*−1_). We chose the following: the mean between consecutive values (*e*_*n*_ + *e*_*n*−1_)/2 for the first variable and the semi-difference (*e*_*n*_ − *e*_*n*−1_)/2 for the second variable (Figs. 6 and 7). Our choice was based on visual clarity and greater trajectory separation (we also tested other embeddings like the above-mentioned time-delay embedding with qualitatively similar results). All reconstructed trajectories in this work were computed directly from the corresponding grand average time series.

#### Normalized trajectories

Normalized trajectories in Fig. 8 were computed by first taking the regular trajectories from 45 and 50 ms perturbation sizes and averaging between them (separately for positive and negative perturbations), then taking each of these and dividing it by its corresponding absolute maximum value (separately for the first and second embedding components). Then we kept the normalized +/−T trajectories as standards and multiplied every trajectory (+/−S, +/−S+/−T, +/−S−/+T) by a factor equal to the ratio between its absolute maximum value and the absolute maximum value of the T trajectories.

#### Areas

Areas in the reconstructed phase space in Fig. 8 are a rough measure of the region where the system is left when the perturbation arrives (step *n* = 0), in units of the normalized trajectories. Areas are ellipses with a center and two radii. The center (which roughly corresponds to the first point of the corresponding normalized trajectory) is the average across subjects of the point in trajectory corresponding to *n* = 0, after pooling 45 and 50 ms perturbation sizes (separately for positive and negative perturbations). The radii are the standard deviation in each component.

### Color blind-friendly plots

We use the ametrine colormap^64^.

### Supporting data and files

The datasets generated and analysed during the current study and the code to plot all figures will be available at the Sensorimotor Dynamics Lab’s website http://www.ldsm.web.unq.edu.ar/spatiotemporal2019.

## Supplementary information

Supplementary Information
