# Supplementary material for: Spatiotemporal perturbations in paced finger tapping suggest a common mechanism for the processing of time errors
Source: Sci Rep. 2019 Nov 28;9:17814. doi: 10.1038/s41598-019-54133-x (PMC6882783; doi:10.1038/s41598-019-54133-x)
Supplement: Supplementary file 1 — Supplementary Information [file 41598_2019_54133_MOESM1_ESM.pdf]

Supplementary Information

.

Spatiotemporal perturbations in paced finger tapping suggest a common mechanism for the processing of time errors

Sabrina Laura López[1,2] and Rodrigo Laje[1,2,*]

[1]Sensorimotor Dynamics Lab, Departamento de Ciencia y Tecnología, Universidad Nacional de Quilmes, Argentina
[2]CONICET, Argentina
[*]rlaje@unq.edu.ar

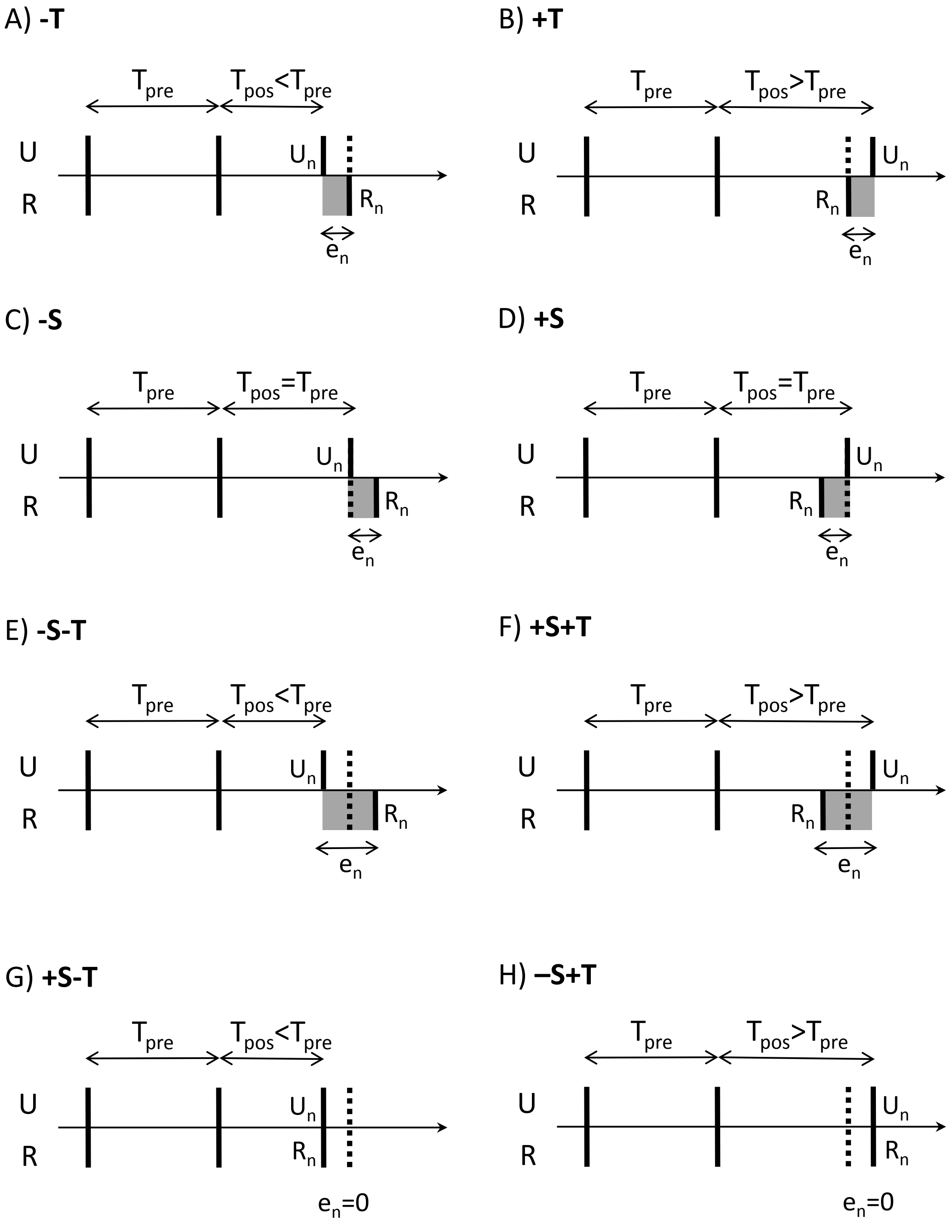

Figure S1: Schematic of perturbations. U = stimuli sequence. R = responses (considering perfect average synchrony for simplicity). Vertical continuous lines: actual occurrences of stimuli and responses. Vertical dashed lines: expected occurrences of stimuli and responses if no perturbation occurs. A vertical dashed line at the level of stimuli means a temporal perturbation occurred. A vertical dashed line at the level of responses means a spatial perturbation occurred. The shaded area indicates the expected average asynchrony at the perturbation step.

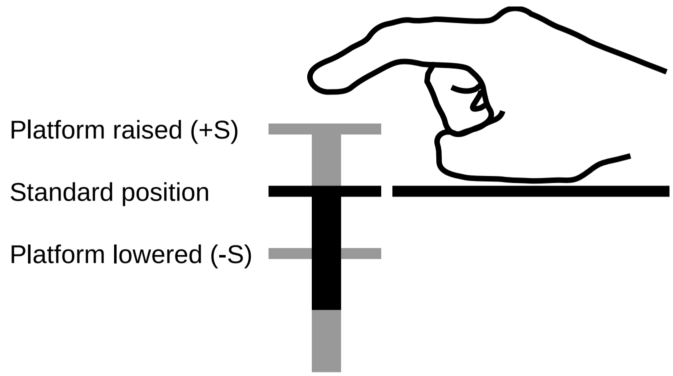

Figure S2: Schematic of platform setup. Spatial perturbations +/-S are performed by raising or lowering a platform by means of a servo motor and a rotating arm (not shown here), and thus advancing or delaying the time of contact, respectively. The force sensor that detects the taps is attached on top of the platform.
